# Using Entropy Maximization to Understand the Determinants of Structural Dynamics beyond Native Contact Topology

**DOI:** 10.1371/journal.pcbi.1000816

**Published:** 2010-06-17

**Authors:** Timothy R. Lezon, Ivet Bahar

**Affiliations:** Department of Computational Biology, School of Medicine, University of Pittsburgh, Pittsburgh, Pennsylvania, United States of America; Fox Chase Cancer Center, United States of America

## Abstract

Comparison of elastic network model predictions with experimental data has provided important insights on the dominant role of the network of inter-residue contacts in defining the global dynamics of proteins. Most of these studies have focused on interpreting the mean-square fluctuations of residues, or deriving the most collective, or softest, modes of motions that are known to be insensitive to structural and energetic details. However, with increasing structural data, we are in a position to perform a more critical assessment of the structure-dynamics relations in proteins, and gain a deeper understanding of the major determinants of not only the mean-square fluctuations and lowest frequency modes, but the covariance or the cross-correlations between residue fluctuations and the shapes of higher modes. A systematic study of a large set of NMR-determined proteins is analyzed using a novel method based on entropy maximization to demonstrate that the next level of refinement in the elastic network model description of proteins ought to take into consideration properties such as contact order (or sequential separation between contacting residues) and the secondary structure types of the interacting residues, whereas the types of amino acids do not play a critical role. Most importantly, an optimal description of observed cross-correlations requires the inclusion of destabilizing, as opposed to exclusively stabilizing, interactions, stipulating the functional significance of local frustration in imparting native-like dynamics. This study provides us with a deeper understanding of the structural basis of experimentally observed behavior, and opens the way to the development of more accurate models for exploring protein dynamics.

## Author Summary

As more protein structures are solved, we are able to perform a more critical assessment of the relationship between protein structure and dynamics, and to gain a deeper understanding of the major determinants of structural dynamics. Here we perform a systematic study on a set of proteins structurally determined by NMR spectroscopy. The dynamics are analyzed using elastic network models and a novel method based on entropy maximization to demonstrate that properties such as contact order and secondary structure do play a role in defining the experimentally observed covariance data. Most importantly, an optimal description of observed cross-correlations requires the inclusion of destabilizing, as well as stabilizing, interactions, stipulating the functional significance of local frustration in imparting native-like dynamics.

## Introduction

Associated with each protein fold is a set of intrinsically accessible global motions that arise solely from the 3-dimensional geometry of the fold and involve the entire architecture. For a number of systems it has been shown that these intrinsic motions play an important role in protein function [1], facilitating events such as recognition and binding [2], [3], catalysis [4]–[6] and allosteric regulation [1], [7], [8]. The time scales of these cooperative motions are usually beyond the reach of conventional MD simulations. They are modeled instead with coarse-grained techniques that omit the finer details of atomic interactions.

The elastic network model (ENM) is an example of a coarse-grained model that has enjoyed considerable success in predicting global dynamics of proteins and other macromolecules. The central idea behind the ENM is that, in the vicinity of a minimum, the potential energy landscape of a biomolecular system can be approximated by the sum of pairwise harmonic potentials that stabilize the native contacts. In the simplest ENM, the Gaussian network model (GNM) [9], each node of the network is identified by an amino acid, and each edge is a spring that provides a linear restoring force to deviations from the minimum-energy structure. The system's dynamics is therefore expressed in terms of the normal modes of vibration of the many-bodied system about its equilibrium state; and dynamical information about the protein, such as the expectation values of residue fluctuations or cross-correlations, is uniquely defined by the network topology.

A few prevalent methods are used for constructing ENMs, but most have at their hearts two underlying assumptions: The springs are all at their rest lengths in the equilibrium (native) conformation, and the force constants decrease with the distance between nodes, among other variables. In the earliest models [9], [10] and the anisotropic network model (ANM) [11]–[13], force constants were taken to be uniform for all nodes separated by a distance less than a specified cutoff distance and zero for greater distances. In parallel, models were proposed in which the force constants decay exponentially [14], [15] or as an inverse power of distance [16], [17], or where stronger interactions are assigned to sequentially adjacent residues [8], [16], [18]. Although such modifications can lead to modest improvements in the agreement between ENM predictions and certain experimental data, there is still no clear “best” method for assigning force constants in an ENM.

A common approach for assessing the performance of ENMs or estimating their force constants has been to compare the ENM-derived autocorrelations of residue motions to the corresponding X-ray crystallographic B-factors or the mean-square fluctuations (MSFs) in residue coordinates observed between NMR models. Because the slow modes have the largest amplitudes, often the focus of study has been a narrow band of the slowest modes. The ENM slow modes have indeed been shown to agree well with those predicted by detailed atomic-level force fields and with experimentally determined dynamics [19], [20]. However, the majority of the dynamical information conveyed by the ENM is contained in the residue cross-correlations, and this information has been largely overlooked during comparisons of ENM results to experimental data. Further, the subtle and complex dynamics of the structures that lie beneath the gross global motions are ignored when only the slowest modes are considered. Mid- and high-frequency modes are predicted with relatively lower confidence by ENMs, but these modes may be important for coordinating the finer motions of the molecule while the slower modes orchestrate its global rearrangements [21]. Finally, while the ENM-based studies have shown that the network topology is the dominant factor that defines the collective modes, especially those in the low frequency regime, there may be other structural properties (e.g. secondary structure, hydrogen bond pattern, distance along the sequence/chain between pairs of interacting residues) that are not accounted for by ENMs but which may provide a more realistic description of equilibrium dynamics, if accurately modeled.

Here we examine the ensembles of structural models determined by NMR for 68 proteins and evaluate for each ensemble the covariance in the deviations of residue-positions from their mean values. We present a technique for optimizing ENM force constants within a pre-defined network topology so as to provide the most accurate representation of the experimentally observed covariance data. Our method is based on the concept of entropy maximization: Briefly, when inferring the form of an unknown probability distribution, the one that is least reliant on the form of missing data is that which maximizes the system's entropy subject to constraints imposed by the available data [22], [23]. This method has been applied to a variety of biological problems, including neural networks [24], gene interaction networks [25], and protein folding [26].

The resulting auto- and cross-correlations in residue fluctuations are used to build an ENM-based model with optimal force constants (OFCs). It can be shown (see [25] and Methods) that when the constraints of the maximization are pair correlations, the probability distribution takes a Gaussian form. Further, the only terms that contribute to the probability distribution are those that correspond to pairs with correlations that are explicitly considered as constraints on the entropy maximization. In terms of the ENM, this means that for a given network topology, there exists a unique set of force constants that exactly reproduces the experimentally observed cross- correlations between all pairs of interacting residues, along with their autocorrelations (or MSFs).

Notably, our technique captures the physical significance of factors such as sequence separation and spatial distance which have been empirically found to influence force constant strengths. Sequence separation is expressed in terms of contact order, i.e., the number of residues along the sequence between two residues that are connected by a spring in the ENM. Further, our analysis benchmarked against a test set of 41 NMR ensembles of proteins suggests additional factors, including hydrogen bond formation and secondary structure type, which should also be incorporated in the ENMs for a more accurate description of experimental data. It also identifies factors that are of little consequence insofar as the collective dynamics near equilibrium conditions are concerned. Amino acid specificity turns out to be one of them; diffuse, overlapping distributions of OFCs are obtained for different types of amino acids, precluding the assignment of residue-specific OFCs. A *m*odified version of the GNM, *m*GNM, that accounts for these factors is proposed and is verified to perform better than existing models especially in reproducing cross-correlations. Finally, the study highlights the importance of higher modes and the role of frustration in protein dynamics, the implications of which are discussed with regard to model development and protein design.

## Results

### Overview of experimental dataset and OFCs

**Figure 1:**
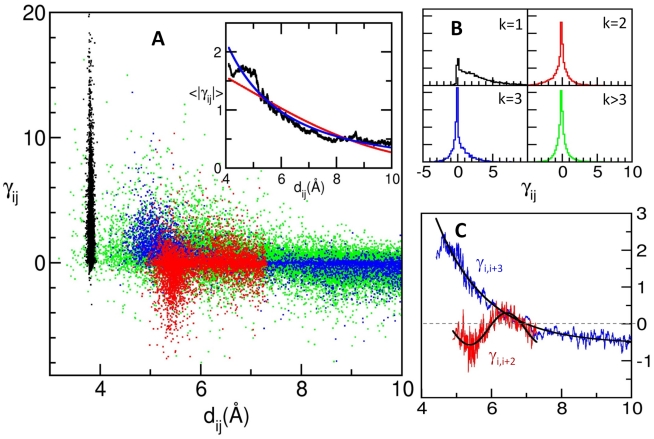
All interactions, colored by contact order. (A) The abscissa displays the distance d_ij_ between residues and the ordinate is the optimized force constant (OFC) deduced from experimental covariance data for the interaction. The black cluster around 3.8Å indicates bonded (*k* = 1) interactions; the red cloud between d_ij_ = 5 and 7.5Å corresponds to second neighbor (*k* = 2) interactions; the blue points indicate *k* = 3 interactions; and the green points in the background indicate all other interactions. Inset shows the trend of average force constant magnitude with distance between nodes (heavy black curve), and two functional fits: red line is 2.26 exp(−d*_ij_* ^2^/46.31); blue line, 31.93/d*_ij_* ^2^. (B) Histograms of the distributions in (A), by contact order. Mean values and standard deviations, μ*_k_*±σ*_k_*, for each curve are μ_1_ = 2.897±3.000; μ_2_ = −0.205±1.035; μ_3_ = 0.385±1.366; μ_>3_ = 0.067±1.124. (C) Trends for the *k* = 2 and *k* = 3 distributions, with the same colors and axes as in (A). The *k* = 2 interactions are fit to a sinusoidal function with extrema around 5.5Å and 6.5Å. The *k* = 3 interactions tend to be positive for small distances (<7Å) and negative for larger distances, decaying exponentially.

The training set of 68 proteins structurally characterized by NMR and deposited in the Protein Data Bank (PDB) [27] (Table S1) contains a total of 252,775 possible pairwise interactions (based on the combination of all pairs of residues), of which 43,118 (17.1%) fall within the 10Å cutoff. Upon optimization, a mean force constant of 6.23 kcal/mol/Å^2^ was found, averaged over all pairs and all proteins. Notably, this value is on the same order as typical uniform ENM force constants [8], [28], and provides an estimate of the strength of generic inter-residue interactions in native folds. To eliminate environment-specific effects and allow for the compilation and comparative analysis of the results for all proteins, we normalized the force constants such that the average force constant magnitude in each protein is unity. The resulting normalized OFCs range from −10.0 to 31.1, in dimensionless units, with a mean of 0.430 and a standard deviation of 1.831. Most (71%) of the force constants have absolute magnitude less than 1.0. Figure 1A displays the distribution of OFCs as a function of the distance *d_ij_* between the interacting pairs of residues *i* and *j*, and colored by contact order *k*. *k* designates the sequential separation between residues *i* and *j*, *k* = 1 corresponding to bonded pairs. The inset in Figure 1A displays the dependence of the average magnitude <|γ_ij_|> on distance.

### Dependence on contact order

A closer examination of the influence of contact order on the OFCs yields the histograms displayed in Figure 1B. Whereas most OFCs are generally small and distributed evenly around zero, those associated with bonded interactions tend to be positive and large, with a mean value of 2.898 and standard deviation of 3.009 (see Figure 1, black dots). These large positive values reflect the almost rigid 3.8Å distance restraints on the backbone pseudo-bonds (virtual C^α^-C^α^ bonds), consistent with the fact that the peptide bond dihedral angle *ω* is confined to the *trans* state, and consequently, in the absence of rotatable bonds the distance between the consecutive α-carbons is almost fixed.

Second-neighbor (*k* = 2) interactions tend to be negative, with mean −0.211±1.436 (red dots in Figure 1A and red histogram in Figure 1B). They obey a unique distance dependence (Figure 1C, red curve), suggesting that 2^nd^ neighbors closer than a certain distance are generally too strained. Likewise, those stretched beyond a certain separation exhibit negative force constants. These interactions add frustration to the system: They tend to favor conformational changes away from the equilibrium structure, but only in a manner that does not violate the more magnanimous *k* = 1 restraints. Taken together, the *k* = 1 and *k* = 2 interactions suggest a flexibility of virtual bond angles, which allows adjacent (first neighboring) residues along the sequence to retain almost rigidly their separation while second neighbors tend to move with respect to each other.

The *k* = 3 interactions (blue dots in Figure 1A), on the other hand, are positive (0.385±1.366) indicating a dynamic correlation between adjacent virtual bond angles. More detailed analysis shows that in this case there is a weak tendency of 3^rd^ neighbors to be destabilized when their distance approaches 10Å (Figure 1C, blue curve). A similar trend is observed in the case of 2^nd^ neighbors, when they approach their maximal separation (∼7.4 Å) allowed by chain connectivity. These observations point to the instability of the conformations that strain the backbone.

### Force constant strengths depend on secondary structure

The *k* = 2 interaction type and strength depend on the distance between residues *i* and *i*+2 (Figure 1C). If the residues are separated by 6Å or less, γ*_ij_* tends to be strong and negative, and the correlation between *k* = 1 and *k* = 2 force constants is −0.386; for distances of more than 6Å, the correlation with *k* = 1 drops to −0.100. This suggests the importance of secondary structure in protein dynamics, which will be our focus next.

**Figure 2:**
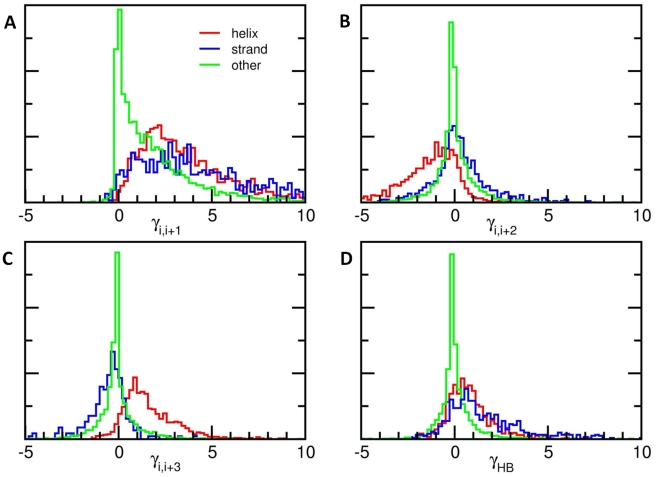
Force constant distributions vary with secondary structure and contact order. Panels A, B and C, respectively, show the force constant distributions for *k* = 1, *k* = 2 and *k* = 3, colored by secondary structure. Red curves indicate force constants between residue pairs in which both amino acids are in α-helices (DSSP code H); blue curves are for force constants between residues in strands (DSSP codes E and B); and green curves are for all other interactions. In α-helices particularly, the *k* = 1 interactions are strong and positive, the *k* = 2 interactions are negative, and the *k* = 3 interactions are again strong and positive. (D) Similar histograms for hydrogen bonding partners. The red curve shows *k* = 4 interactions in α-helices, the blue curve shows force constants between hydrogen bonding partners in strands, and the green curve shows all other interactions for *k*>4.

In helices, second neighbors tend to be separated by about 5.47±0.20Å, compared to 6.66±0.41Å in strands. As can be seen from the red curve in Figure 1C, the former separation coincides with the minimum (i.e., largest negative value) in the OFC curve, which is also consistent with the red histogram displayed in Figure 2B for α-helices. The positioning of α-carbons *i* and *i+2* along an α-helical turn requires the dihedral angles *ϕ* and *ψ* on both sides of C^α^ *_i_* to assume narrowly distributed values in the Ramachandran space and entails relatively tight packing of side chains, which may not be sufficiently stable *per se*, unless stabilized by hydrogen bonds formed between the adjoining residues on both sides. No such effect is discerned in 2^nd^ neighboring residues on β-strands, given that the corresponding dihedral angles are more broadly distributed, and the backbone conformation allows for favorable interactions between every other side chain.

Notably, 3^rd^ neighbors on β-strands tend to exhibit negative OFCs (Figure 2C). The *C^α^_i_-C^α^_i+3_* distance of 8.796±1.408 Å falls in the regime of negative force constants (see the blue curve in Figure 1C). In the case of helices, third neighbors are located at a distance of 5.230±0.531 Å, and experience favorable interactions on a local scale (Figures 1C and 2C). The flexibility of the β-strand *k* = 3 contacts and the rigidity of the β-strand *k* = 1 and *k* = 2 contacts suggests that strands have a propensity for twisting motions.

### OFCs are consistent with hydrogen bond formation patterns

Hydrogen bond formation is also found to have a strong influence on the OFCs. Using the DSSP [29] algorithm, we determined secondary structures for residues in our dataset and found that the interactions between hydrogen-bonded residues tend to be larger than those between residues that are not hydrogen-bonded (see Figure 2D), which strongly supports the physical realism of the derived OFCs. In α-helices, the average OFC for *k* = 4 interaction representative of hydrogen-bonded residues on consecutive turns is 0.962±1.341, compared to 0.137±1.008 for all other *k* = 4 interactions. Similarly, interactions between hydrogen-bonded partners in extended strands or isolated β-bridges have values around 1.801±2.321, compared to 0.412±1.817 for other interactions, thus more than counterbalancing the destabilizing interactions between 3^rd^ neighbors. In both cases, the distributions for hydrogen-bonded and non-hydrogen-bonded interactions overlap significantly but are distinct, with Kolmogorov-Smirnov [30] probabilities of less than 10−^44^. This sensitivity to atomic-level details is missing in many coarse-grained ENMs, but it is an essential component of the potential energy.

### Interplay between destabilizing and stabilizing interactions on a local scale

Clearly, despite the existence of destabilizing interactions on a local scale, the overall structure is stable, i.e., the native structure is a global energy minimum (as also confirmed mathematically; see Methods) because these destabilizing pairwise interactions are more than counterbalanced by other stabilizing interactions. For example, there is a weak (−0.274) anti-correlation between the *k* = 1 and *k* = 2 force constants, and more significant anti-correlations between *k* = 2 and *k* = 3 (−0.689) and between *k* = 4 and *k* = 5 (−0.614) (See Table 1). In particular, when residues *i* and *i*+2 are in helices, the force constants corresponding to the interactions between first and second neighbors exhibit a correlation of −0.641 (see also Figure S1). The third and fourth neighbors on α-helices, on the other hand, are distinguished by their strong stabilizing interactions (Figure 2C and D). Similar effects occur between 2^nd^ and 3^rd^ neighbors in β-strands, and in all cases hydrogen bonds appear to make significant contributions to the overall stability. The presence of these (anti)correlations suggests that on a local scale there is a subtle balance between favorable and unfavorable interactions that is instrumental in determining the marginal stability of the molecule as well as its collective motions about the equilibrium structure.

**Table 1:** Correlations between optimized force constants associated with contact orders of k≤5, indicative of compensating interactions between near neighbors along the sequence.

	*k* = 1	*k* = 2	*k* = 3	*k* = 4	*k = 5*
***k*** ** = 1**	1.000	**−0.274**	**0.206**	**0.259**	**−0.285**
***k*** ** = 2**	−0.641 (−0.193)	1.000	**−0.689**	**−0.169**	**0.256**
***k*** ** = 3**	0.610 (−0.353)	−0.578 (−0.562)	1.000	**0.251**	**−0.437**
***k*** ** = 4**	0.206 (−0.100)	0.042 (−0.210)	0.307 (−0.128)	1.000	**−0.614**
***k*** ** = 5**	−0.340 (−0.189)	0.082 (0.163)	−0.454 (−0.201)	−0.787 (−0.500)	1.000

The upper triangle indicates results for all residues (written in boldface), and the lower triangle indicates results for pairs of residues in helices (strands) only. See Methods for calculation details.

### Force constant strengths are not residue-specific

We analyzed the dependence of the OFCs on amino acid type and coordination number. The distribution of force constant strengths exhibit some variations by amino acid type as can be seen from the heights and widths of the distributions in Figure S2, but there is no specific correlation of force constant values with amino acid type. Although each amino acid has a unique distribution of force constant strengths, all of these distributions overlap to a large extent, so that accurately predicting interaction strength based on amino acid type is not possible. This observation agrees with the longstanding argument that the global dynamics of solvated proteins are structure-based, and not sequence-based. We note that the insensitivity of force constants to amino acid type does not imply that all contacts contribute equally to the free energy, but that the deviations from their equilibrium positions experience comparable resistance. In terms of energy function, the *depths* of the energy minima may dependent on amino acid types, but the *curvatures* of the energy profiles near the minima do not exhibit residue-specific features at this coarse-grained level of representation.

### Dependence on packing density

As was seen through the large values of the bonded interactions, physical constraints directly impact the interaction values. We therefore expect the OFCs to be greatest in magnitude for the spatially constrained residues in the protein interior, and the mean-square fluctuations to decrease with the coordination number. Indeed, there is a modest (0.508) correlation between the magnitudes of the bonded interactions and the coordination numbers of the nodes they join. There is a stronger (−0.582) (anti)correlation between the coordination number and self-interaction, and a very strong (−0.909) one between a residue's self-interaction and the sum of its interactions with its first neighbors. The weight of the node, defined as the sum of the magnitudes of its edges, relates inversely to its MSF in much the same way as the degree of a node in GNM relates to its MSF (Figure S3).

### Dependence on physical distance

Although the force constants vary in value at all distances, we were curious to examine in more detail whether there exists an underlying trend that describes the force constant magnitude as a function of distance between residues. We calculated the average absolute magnitude of the force constants as a function of residue separation (see Figure 1A, inset) and examined the functional form of this distance dependence. Using a function of the form 
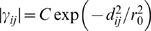
 as proposed by Hinsen [14], we find the highest correlation of only 0.339 when the distance *r_0_* is 6.805Å, which is about twice the proposed value of *r*_0_ = 3.0Å for non-bonded force constants. Fitting the average magnitude to a function of the form 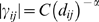, we find the best fit (cc = 0.356) using an exponent of α = 1.953, which is remarkably close to the exponent α = 2 suggested by Jernigan and coworkers [17]. Although the trend is for the average magnitude of force constants to decay with distance between nodes, the correlations are not very strong and the abundance of noise in the force constants prohibits the identification of a definitive function with which they universally decay. Figure 1C shows that the distance dependence also varies with contact order.

### Comparison to GNM

We compared the collective dynamics calculated with GNM to those found via OFCs (shortly referred to as OFC-GNM), with regard to the level of agreement achieved with experimental data. The computed covariance matrix contains three types of elements: diagonal, interacting (nodes joined with an edge) and non-interacting. Diagonal elements are representative of the MSFs of individual residues, and off-diagonal terms represent the cross-correlations between the fluctuations of pairs of residues. Table 2 summarizes the level of agreement of the two methods with the experimentally observed covariances. Notably, the optimized model provides a more accurate description of not only MSFs and cross-correlations between connected nodes, but also the cross-correlations between pairs of residues that are located farther apart in the structure. As shown in Table 2, experimental covariances between non-interacting residues have a correlation of 0.759 with the covariances predicted by OFC-GNM, compared to −0.014 for GNM.

**Table 2:** Correlations between experimentally observed covariances(*)with those predicted by GNM with uniform force constants, and the GNM with optimized force constants (OFC-GNM).

Correlations with experiments\ENMs		GNM	OFC-GNM
Autocorrelations	MSFs	0.743±0.145 (0.734±0.203)	1.000 (0.997±0.007)
Cross-correlations	All	0.578±0.114 (0.365±0.169)	0.967±0.020 (0.904±0.058)
	Interacting	0.527±0.195 (0.534±0.195)	1.000 (0.994±0.008)
	Non-interacting	−0.014±0.187 (0.028±0.169)	0.759±0.148 (0.746±0.153)

(*) Based on 3649 NMR models from 68 proteins (see Table S1).

Values in parenthesis indicate the level of agreement when only the top 5 modes are considered.

One attractive feature of GNM is its ability to provide results that are robust against minor changes in structure or network topology. To test the resilience of OFC-GNM dynamics, we set small force constants identically to zero and re-calculated the covariance matrix. When the smallest 5% and 10% of the interactions are discarded, the correlation between OFC-GNM and experiment drops from 0.967±0.020 to 0.407±0.443 and 0.238±0.347, respectively. Unlike the GNM, the optimized model is therefore quite sensitive to the existence or loss of weak interactions. We also examined the robustness of the modes in the low frequency regime. The values in parentheses in Table 2 shows that the top ranking five modes computed with the OFC-GNM yield good agreement with their experimental counterpart, whether the GNM cross-correlations exhibit a considerable decrease in their level of agreement with experiments.

### GNM predictions can be improved using additional information

We briefly investigated whether the trends observed in the optimized force constants can be used to create a more effective ENM. Using a separate set of 41 proteins (Table S2), we tested the effects of incorporating bonded interactions, second neighbor interactions and hydrogen bonding into the ENM. The results, summarized in Table 3 and Table S3, indicate that including these properties mildly improves the agreement of the ENM with observed covariances for the test set. We obtained the best agreement when bonded interactions and hydrogen bonded interactions are increased in magnitude and second-neighbor force constants are negative. One set of parameters for this model, which we refer to as modified GNM or mGNM, is given in Table 3.

**Table 3:** Correlations between various ENM-predicted covariances and those observed in NMR experiments(a).

Model(b)		cc (RMSF)	cc (off-diagonal)	cc (all covariance)
1	U (GNM)	0.689±0.188	0.402±0.163	0.553±0.135
2	D	0.724±0.177	0.431±0.150	0.555±0.136
3	U+γ_(1)_	0.722±0.184	0.438±0.142	0.544±0.134
4	D+γ_(1)_	0.706±0.191	0.416±0.129	0.502±0.128
5	U+γ_(1)_+γ_(2)_	0.720±0.188	0.448±0.150	0.558±0.140
6	D+γ_(1)_+γ_(2)_	0.726±0.192	0.452±0.142	0.545±0.138
7	U+γ_(1)_+HB	0.731±0.179	0.453±0.146	0.565±0.136
8	D+γ_(1)_+HB	0.724±0.182	0.430±0.132	0.521±0.129
9	U+γ_(1)_+γ_(2)_+HB	0.727±0.184	0.465±0.154	**0.579±0.142**
10	D+γ_(1)_+γ_(2)_+HB (mGNM)	**0.738±0.190**	**0.472±0.147**	0.570±0.141

(a) Results obtained for the test set of proteins listed in Table S2.

(b) Symbols used are: U – Uniform (γ = 1) force constant; D - distance-dependent (γ = 1/d^2^) force constant; γ_(1)_ – Nearest neighbor interactions are increased by a factor of 10; γ_(2)_ – Second neighbor interactions are changed by a factor of −1 in U models or −5 in D models; HB – Interactions between residues joined by backbone hydrogen bonds are increased by a factor of 10.

Values by protein can be found in Table S3.

## Discussion

At present, there are copious NMR and X-ray data available from which we can extract information on protein equilibrium dynamics, and the current state of molecular dynamics is such that one can likewise approximate equilibrium ensembles of small proteins *in silico*. By developing coarse-grained models that reproduce these dynamics, we are able to deepen our understanding of the factors that influence protein folding and function.

In the present analysis we selected to use NMR data that provide conformational ensembles based directly on experiments, but any covariance data could have been used, in principle. The REACH algorithm [31] identifies effective ENM force constants through an inversion of a covariance matrix derived from MD simulations. Similarly, the heteroENM [32] utilizes an iterative algorithm to similarly fit the force constants with MD-derived covariances. The advantages to using MD-derived covariances are precision and flexibility. Because the locations of all atoms in an MD run are known to machine precision in each simulation frame, the covariance between even the most distant atoms, such as those separated by several nanometers, can be exactly calculated within the context of the simulation. Further, MD simulations permit *in silico* alterations to the system under study, allowing one to find effective force constants that are specific to any environment that can be simulated. This is a boon in particular to those who wish to study the global dynamics and interactions of multiple large molecules. On the other hand, there are some shortcomings of MD that make it an unattractive option for developing an ENM. First, MD is itself a theoretical model, and the performance of any MD-based ENM is limited by the accuracy of the force field: Inaccurate MD results beget inaccurate ENM results. Second, MD is stochastic in nature, insofar as simulations of identical systems starting from different initial states may produce different results due to sampling inaccuracies. Finally, MD is generally applicable only for short (<1µs) simulations. Covariances calculated over a short time should not be assumed to remain valid when the timescale is increased by several orders of magnitude.

Amino acid covariances are calculated here from experiments, specifically NMR structural data. A few well-studied proteins have been crystallized in multiple states – such as those bound to different ligands – allowing residue covariances to be calculated from X-ray data. Although a growing body of work suggests that functional states assumed by the proteins under different conditions are captured in multiple crystal structures [33]–[36], such multiple X-ray crystallographic structures have been determined for a few well-studied proteins only, and in most cases proteins crystallized in diverse states may not be representative of the native ensembles of conformations accessible to the protein. A more abundant source of protein conformational ensembles is NMR data. The use of various NMR techniques in determining solution dynamics of proteins has been reviewed extensively (see, for example, [37], [38]), and a number of techniques have been proposed for inferring native-state protein ensembles from NMR data [39]–[43]. Covariances calculated from NMR ensembles have been shown to agree well with MD [44], X-ray B-factors [45], [46] and covariances between multiple crystal structures [33]–[36]. NMR data are not, however, without their shortcomings: NMR ensembles may be affected by the sparsity of data and conformational variations found in solution, and as such they necessarily contain noise and do not purely reflect the native state ensemble. As the NOE intensities that are used to define structures decay rapidly with interatomic distance, long-ranged interactions are a likely source of noise in NMR covariance data. Force constant optimization methods that rely on full covariance data [31], [32] retain this noise. We were able to identify the major determinants of the effective force constants that describe the collective dynamics of proteins by resorting to a rigorous entropy maximization procedure that addresses such uncertainties.

Strikingly, a subtle interplay between stabilizing and destabilizing interactions has been disclosed, which depends on contact order, secondary structure and hydrogen-bond-formation properties. Although all of the proteins that we have analyzed are relatively small, the physical basis of the factors impacting force constant strength leads us to believe that our results hold for larger proteins as well.

The OFCs are derived from existing structural data, and in this respect our work is similar in spirit to the extraction of knowledge-based potentials from known structures [47]–[53]. The present study differs, however, in four ways: First, previous studies aimed at evaluating the effective potentials of mean force that determine the equilibrium state/energetics of native structures, and they were used in evaluating folded or docked conformations. Here, the goal is to assess the effective force constants that determine the collective fluctuations away from the equilibrium state, which are used in evaluating the equilibrium dynamics. Second, the training dataset consists of distinct proteins' structures in the former approach, whereas here ensembles of conformations corresponding to a given protein are analyzed. Third, the former group of studies counts the probabilistic occurrences of inter-residues pairs (or pair radial distribution functions) to derive potentials of mean force using inverse Boltzmann law; here, the departures in coordinates from their mean values are examined, and optimal spring constants are evaluated from an entropy maximization scheme, which is appropriate for sparse data. Fourth, the knowledge-based potentials evaluated in previous studies are residue-specific, whereas the OFCs show no significant dependence on amino acid type. This final observation is in accord with the concept that amino acids influence the fold, and the fold influences the dynamics.

In our calculations we intentionally used a slightly longer cutoff distance (10Å) than those determined to optimally reproduce B-factors (7–8Å) [19], [54]. Our reasoning was that, if a shorter cutoff distance is better, then force constants for residues that are far from each other will tend to be close to zero. Although we find that the average magnitude of the force constants decays with distance, we do not find that the force constants all drop sharply to zero after some distance. GNM consistently predicts global protein motions that agree with experimental observations, using a uniform force constant. It would therefore not have been unexpected to find that the OFCs tend to cluster about a single non-zero value. Instead, we find that the OFCs adopt a range of values centered about zero, and that the strongest indicators of force constant strengths are contact order and backbone hydrogen bond formation propensities.

**Figure 3:**
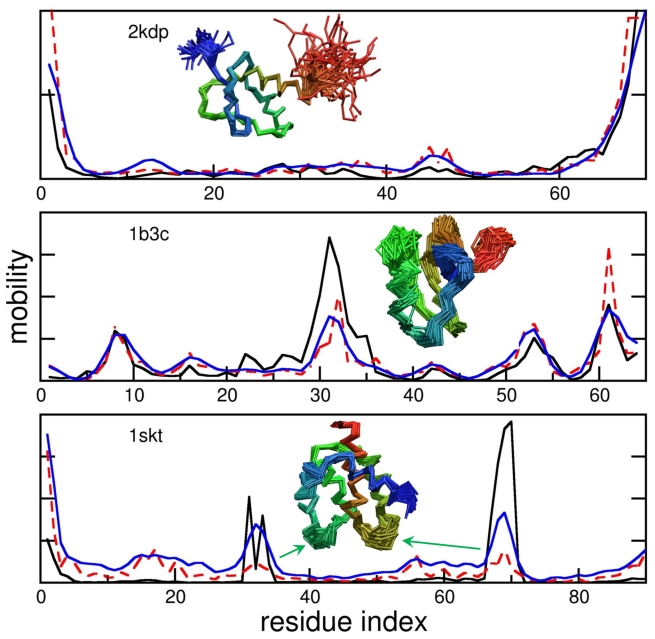
GNM-predicted motions display a range of overlaps with observed mobilities. The panels display the mobility profiles for three example proteins from our test set to illustrate the various levels of agreement observed between theory and experiments. The curves are calculated from the first five modes of the covariance matrix deduced from NMR experiments (solid black lines), the five slowest GNM modes (dashed red lines) and the five slowest mGNM modes (solid blue lines). Insets are cartoons of the NMR ensembles for the three proteins, colored blue to red from the N-terminus to the C-terminus. An example of good agreement between GNM and observed covariances is the histone deacetylase complex protein 2kdp (top panel), for which the GNM accurately predicts high mobility at the termini. The correlation coefficient (cc) between theory and experiments is 0.91 in this case for both GNM and mGNM, due in large part to the motion at the protein termini. Average agreement of 0.67 is seen in the scorpion neurotoxin 1b3c, for which GNM predicts excessive motion near the C-terminus and under-predicts motion of the loop around residue 32, shown in green in inset cartoon. When mGNM is used, the sharp changes in the mobility profile are smoothed and the correlation increases to 0.79. In the calcium binding protein1skt (bottom), the GNM predicts motion at the N-terminus, whereas the NMR ensemble shows higher variation around the two turns around residues 33 and 69 (green arrows). The mGNM improves agreement by increasing mobility around these turns. The correlation between theory and experiments is increased from 0.31 to 0.57 upon adopting the mGNM instead of the GNM (with uniform force constants).

The difference between the predictions of the GNM and observed protein motions is illustrated in the three examples of Figure 3, selected from the test set (Table S2). The three curves therein represent the MSFs of residues based on five slowest modes derived from NMR data (black, solid), predicted by the GNM (red, dashed), and predicted by the mGNM (blue curve). As the GNM is based entirely on the protein's folded topology, it tends to instill the most motion in the least connected nodes, e.g., chain termini or the most exposed loop regions. However, the size of the motion may depart from those indicated by NMR models, and mGNM tends to yield a better agreement with NMR data. Application to the complete test set of NMR ensembles confirmed that the correlation with experiments is improved even when contact order, distance dependence and hydrogen bonding are incorporated into the GNM without laboriously optimizing the force constants (Table 3). The fact that these physically meaningful effects emerged independently from our entropy maximization calculations validates our approach to some extent. Less expected was the prominence of negative force constants.

Overwhelmingly, the methods of ENM construction rely on two assumptions that guarantee physically plausible behavior, but which may be unwarranted. The first is that all springs are at their rest lengths in the equilibrium conformation, and the second is that all spring constants are positive. Taken together, these assumptions are sufficient, but not necessary, to guarantee that any deformations will increase the system's energy. Our optimization procedure naturally produces interactions that are physically equivalent to springs of negative force constant, but so long as the interaction matrix remains nonnegative definite, the system is in a stable equilibrium and negative force constants are acceptable. The existence of negative force constants reflects the implicit frustration of folded proteins; the backbone restrains the protein to certain compact folds, and not all native state contacts are guaranteed, nor should be expected, to be favorable. Negative force constants make the structure prone to certain deformations that may not be preferred when all force constants are positive. Frustration in proteins results in a rough free-energy landscape that gives rise to folding intermediates and alternative conformations [55]–[58], and calculations involving Go-like potentials, or knowledge-based potentials [49] reveal the requirement to include both stabilizing and destabilizing interactions for an accurate assessment of the folding behavior or stability of proteins. The balance between attraction and repulsion endows proteins with both the sensitivity and the stability that are prerequisite for proper function [59]. We find that the (*i*, *i*+2) interactions are the most likely to be at a local maximum, promoting a change in the angle between (*i*, *i*+1) and (*i*+1 *i*+2) pseudobonds.

When we include factors such as hydrogen bonds and negative *k* = 2 force constants in the GNM, the improved agreement comes in the off-diagonal components of the predicted covariance matrices. Cross-correlations are often overlooked when assessing ENM predictions, but they are essential because they carry information on how the molecule moves as a whole. The autocorrelations that indicate how much individual residues move are each the sum of positive terms and are necessarily dominated by the slower modes. The cross-correlations, on the other hand, are sums of positive and negative terms and are therefore susceptible to the influence of higher modes. Slight modifications to the GNM, such as those that we have introduced in mGNM, do not perturb the network enough to significantly alter the slow modes (Figure 3), but their effects are captured in the higher modes.

Although the slowest modes get the most attention because of their prevailing role in determining the molecule's global motions, the high-frequency modes have shown to be important for identification of conserved residues and folding cores [60]–[63]. Mid- to high-frequency modes are also crucial to all aspects of protein behavior. Allosteric transitions have been shown to occur largely along the slowest modes, but higher modes are essential for the complete transition [64]. Similarly, a protein's response to external perturbations [28] is dependent on all modes, not only the slowest few. An ENM that accurately captures all modes has an enhanced ability to predict large-scale conformational changes, and our technique opens the door to developing better ENMs based on experimental data.

**Figure 4:**
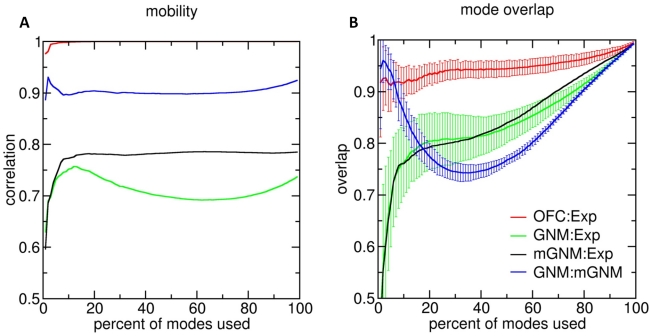
The effect of non-uniform force constants is manifested in the mid-range modes. The curves compare mobility (A) and mode overlap (B) of models as a function of the fraction of modes used. Black, green, and red curves compare the modes of the inverse covariance matrices from experiments to those obtained using mGNM, GNM, and optimized interactions (OFCs), respectively. The blue curves compare GNM modes to mGNM modes. For clarity, some error bars have been omitted. See text for details.

Figure 4 shows pairwise comparisons of the eigenspaces spanned by the slowest modes of various models. Panel A shows the correlation of mobilities as a function of the fraction of modes used in the comparison, and panel B shows a similar plot of the overlap of the eigenspaces (see Methods). The green and black curves relate the GNM and mGNM, respectively, to the experimental covariance matrices. The average mobility correlation of GNM with the experimental covariances peaks at 0.76 when 12% of the modes are considered and then falls as more modes are taken into account, indicating that the predicted modes in the mid-to-high frequency range introduce errors manifested by departures from experimental data. The modified GNM does not exhibit this decline, but remains steady even as higher modes are considered, indicating that the higher modes of the mGNM do not adversely affect the predicted mobility of the system. Comparison of GNM to mGNM (blue curves) shows that the slowest 2% of modes of these models are highly overlapping, but that the similarity decreases as more modes are considered. The modifications of mGNM therefore do not affect the slowest mode, which is presumably determined by the fold topology, but they change the shapes of higher modes.

Interestingly, the overlaps of the GNM and the mGNM with the modes of the covariance matrix are almost identical (compare green and black curves, panel B), suggesting that, despite the improved agreement in mobility, the modifications that we have made to the mGNM still fail to precisely capture the system's overall dynamics. Although some additional improvement may be gained by fine-tuning the parameters of the mGNM (last line, Table 2), the similarity in slow modes of GNM and mGNM once again indicates that fold topology has the dominant influence on the mode shapes.

## Methods

### Protein sets

For our training set, we start with a set of 68 proteins (Table S1), each of which has at least 40 NMR structures available. The proteins in our set have between 43 and 151 residues. For each protein we calculate the mean structure from the NMR ensemble, and we select as a representative structure the NMR model that has lowest root-mean-square deviation (RMSD) from the mean. The test set consists of 41 proteins (Table S3), each having at least 40 NMR models and no fewer than 50 residues.

### Assessment of optimal force constants

We seek to determine the pairwise interactions that optimally describe observed covariances between residues while minimizing the assumptions about the form of missing data. For this, we turn to the principle of maximum entropy, which states that when inferring the form of an unknown probability distribution from a limited number of samples drawn from the distribution, the method that is minimally reliant on the form of missing data is entropy maximization. Here the central idea is outlined in terms of the GNM.

Consider a protein of *N* residues for which *m* structures are known (e.g., *m* models deposited in the PDB for a given protein resolved by NMR spectroscopy). The position of residue *i* in structure *k* is given by the vector, 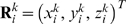, the average position of residue *i* in all structures that have been optimally superimposed (to eliminate external degrees of freedom) is defined as 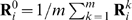, and the vector displacement of residue *i* in structure *k* from the average is 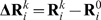. In the GNM, we replace the vector displacement **ΔR** *_i_* with the scalar displacement Δ*r_i_*, which is defined such that 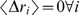 and 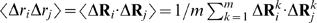.

Now define the set π of *q* pairs of residues such that for all pairs 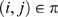 we know the covariances 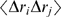, but for pairs 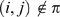 we do not know 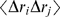. We seek the probability distribution that produces the known covariances while remaining minimally presumptive about the form of missing information. According to Jaynes [22], [23], this is the distribution that maximizes entropy subject to the constraints that some pair covariances are known and must be reproduced.

Defining the *N*-component vector, 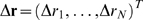, the probability distribution that we seek is *ρ*(**Δr**), and it has the properties
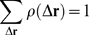
(1)

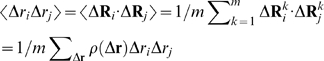
(2)We define the entropy 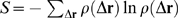, and impose the above constraints as Lagrange multipliers:
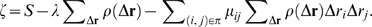
(3)Maximizing ζ with respect to *ρ*(**Δr**), we find
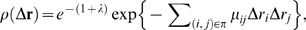
(4)or, defining Z = e^1^+^λ^. and the matrix **K** with elements K_ij_ = μ_ij_,
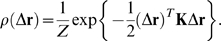
(5)Direct integration leads to the result
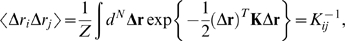
(6)which is the well-known relationship between covariances and pair interactions. The probability distribution in Equation 5 is of the same Gaussian form as the probability distribution from GNM [9], but with the interaction matrix **K** replacing the product of the spring constant γ and the Kirchhoff matrix **Γ**. Thus, the off-diagonal elements of **K** correspond to the negative spring constants: K_ij_ = −γ_ij_, where γ_ij_ is the force constant of the interaction between residues *i* and *j*. We are claiming knowledge for the covariance information of only the *q* residue pairs in the set π, so **K** cannot be found through the simple inversion of the covariance matrix. The matrix **K** has a well-defined form: the elements 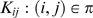 are the Lagrange multipliers that have imposed the above constraints on the covariance and may therefore be different from zero; the elements 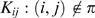 are identically zero. Mathematically, this means that there are no constraints on the covariances of pairs 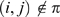. We then have partial information for both **K** and **K**−^1^: The elements 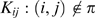 and 
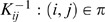
 are known, and the elements 
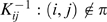
 and 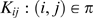 are to be determined. The solution can be found through an *N*-dimensional minimization as follows. Consider the function
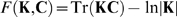
(7)of two symmetric square matrices **K** and **C**. Differentiation with respect to each element of **K** reveals that there exists a single minimum at
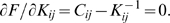
(8)Because *C_ij_* is undefined for all 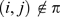, we can allow 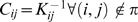, automatically satisfying the minimization condition for elements not in π. The remaining elements of **K** can be found by starting with a matrix of the general form of **K** and iteratively adjusting the non-zero elements against the gradient given in Eq. 8 until the minimum is reached. Optimization is achieved when 
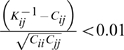
 for all interactions. This criterion appears to be sufficiently strict: Reducing the optimization constant from 0.01 to 0.005 changes the spring constants by less than 1%, on average. The optimization is somewhat computationally intensive: Each step requires an O(*N* ^3^) matrix inversion, and the minimization completes after about 10^4^ steps, making this technique best-suited for small proteins.

It is noteworthy that only those interactions corresponding to known covariances are optimized, and the rest remains zero. This result stems from the application of entropy maximization. Whereas many networks are capable of exactly accounting for the covariance information in the *q* known interactions, this is the only one that does so without prior assumptions about other covariances. Each pair interaction carries information on the covariance of two of the *N* nodes, so a network of more than *q* interactions carries information on more than *q* covariances. Nevertheless, all covariances can be calculated with the resultant network. Those covariances that are not known *a priori* and included in the calculation simply result from the optimized interactions. The matrix **C** is nonnegative definite by construction, and its inverse **K** is therefore also nonnegative definite. As a result, no deviation from the native state conformation can lower the system's energy.

The interaction matrix **K** has the dimensions of Å^−2^, and physical values for the force constants can be determined by multiplying by 3*k_B_*T, where *k_B_* is the Boltzmann constant and T is the temperature. Using this conversion, the OFCs vary between −1686 kcal/mol/Å^2^ and 3868 kcal/mol/Å^2^, with a mean of 6.23 kcal/mol/Å^2^. When **K** is scaled by a scalar constant, γ, its corresponding covariance matrix is scaled by γ^−1^. Thus, the mean element magnitude of the covariance matrix affects the magnitudes of the elements of the interaction matrix, such that large covariances tend to produce weak interactions. The experimental conditions under which the structures are solved influence the magnitudes of the covariances, and therefore also influence the magnitudes of the effective force constants. To reduce the bias on force constants caused by environmental specificity, the OFCs for each protein are scaled by the mean magnitude of the non-zero off-diagonal interactions in that protein.

### GNM

In the GNM, each residue is a node of the network and is represented by its C^α^ atom. Nodes that are within a cutoff distance, *R_c_*, are considered connected via an elastic edge. Typical values of *R_c_* are between 7Å and 10Å. Using the *N*-dimensional column vector, 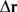, of displacements of the nodes from their equilibrium positions, the potential energy is found to be 
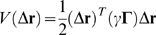
, where γ is a uniform force constant assigned to all interactions, and **Γ** is the Kirchhoff adjacency matrix, with off-diagonal elements Γ*_ij_* = −1 if nodes *i* and *j* are in contact and Γ*_ij_* = 0 otherwise. The diagonal elements of **Γ** are such that the sum over all elements in any row or column is identically zero. The elements of the covariance matrix predicted by the GNM are related to **Γ** as 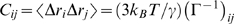.

### Mode overlap

If **U** and **V** are two sets of normal modes for an *N*-dimensional system under different models, then we define the overlap of the first *m* modes of the models as 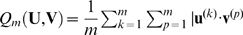, where **u** ^(*k*)^ and **v** ^(*p*)^ are the *k^th^* and *p^th^* slowest modes of **U** and **V**, respectively. *Q_m_* ranges from 0, if none of the space spanned by the slowest *m* modes of **U** can be projected onto the first *m* modes of **V**, to 1, if the two spaces overlap exactly.

### Correlation between force constants

The force constant between residues *i* and *i*+*k* is 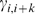. The correlation coefficient between force constants corresponding to different contact orders is calculated as follows. First, for a contact order *n<k*, we define 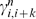 as the average force constant for all pairs between *i* and *i+k* that have a contact order of *n*:
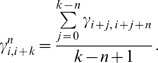
(9)The correlation between force constants 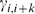 and 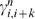 is then
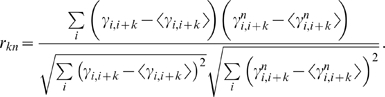
(10)
Table S2 lists such correlations for contact orders in the range 1≤*k*≤5.

## Supporting Information

Figure S1 Distribution of the force constants corresponding to non-bonded interactions of twenty different types of amino acids. Axes are identical in all plots. Mean values and standard deviations are listed in each case.
(1.66 MB TIF)

Figure S2 Scatter plots of *k* = 2 force constants against *k* = 1 force constants for helices (red circles) and strands (blue squares).
(1.31 MB TIF)

Figure S3 Relationship between mean square fluctuations and inverse node weight. In GNM (red circles) the weight of a node is the number of its edges, ni. In OFC-GNM (blue squares), the edge weight is the sum of the magnitudes of all its edges. The correlations with the linear fits shown are 0.416 and 0.670, respectively.
(1.15 MB TIF)

Table S1 Training set proteins
(0.06 MB DOC)

Table S2 Test set proteins
(0.05 MB DOC)

Table S3 Test set mGNM results by protein
(0.08 MB DOC)
