# Supplementary figures and images for: Using Entropy Maximization to Understand the Determinants of Structural Dynamics beyond Native Contact Topology
Source: PLoS Comput Biol. 2010 Jun 17;6(6):e1000816. doi: 10.1371/journal.pcbi.1000816 (PMC2887458; doi:10.1371/journal.pcbi.1000816)

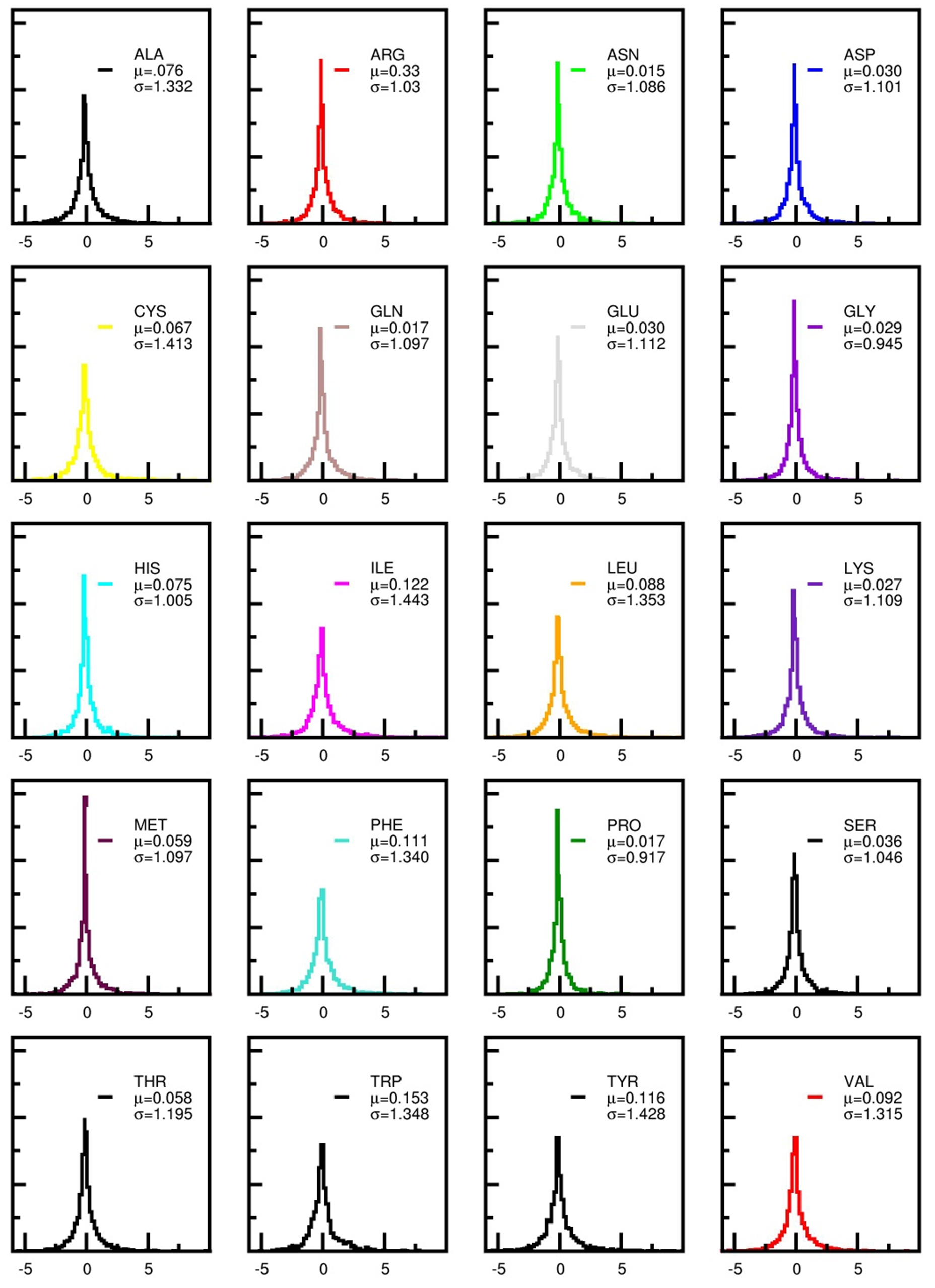

Supplement: Figure S1 — Distribution of the force constants corresponding to non-bonded interactions of twenty different types of amino acids. Axes are identical in all plots. Mean values and standard deviations are listed in each case. (1.66 MB TIF) [file pcbi.1000816.s001.tif]

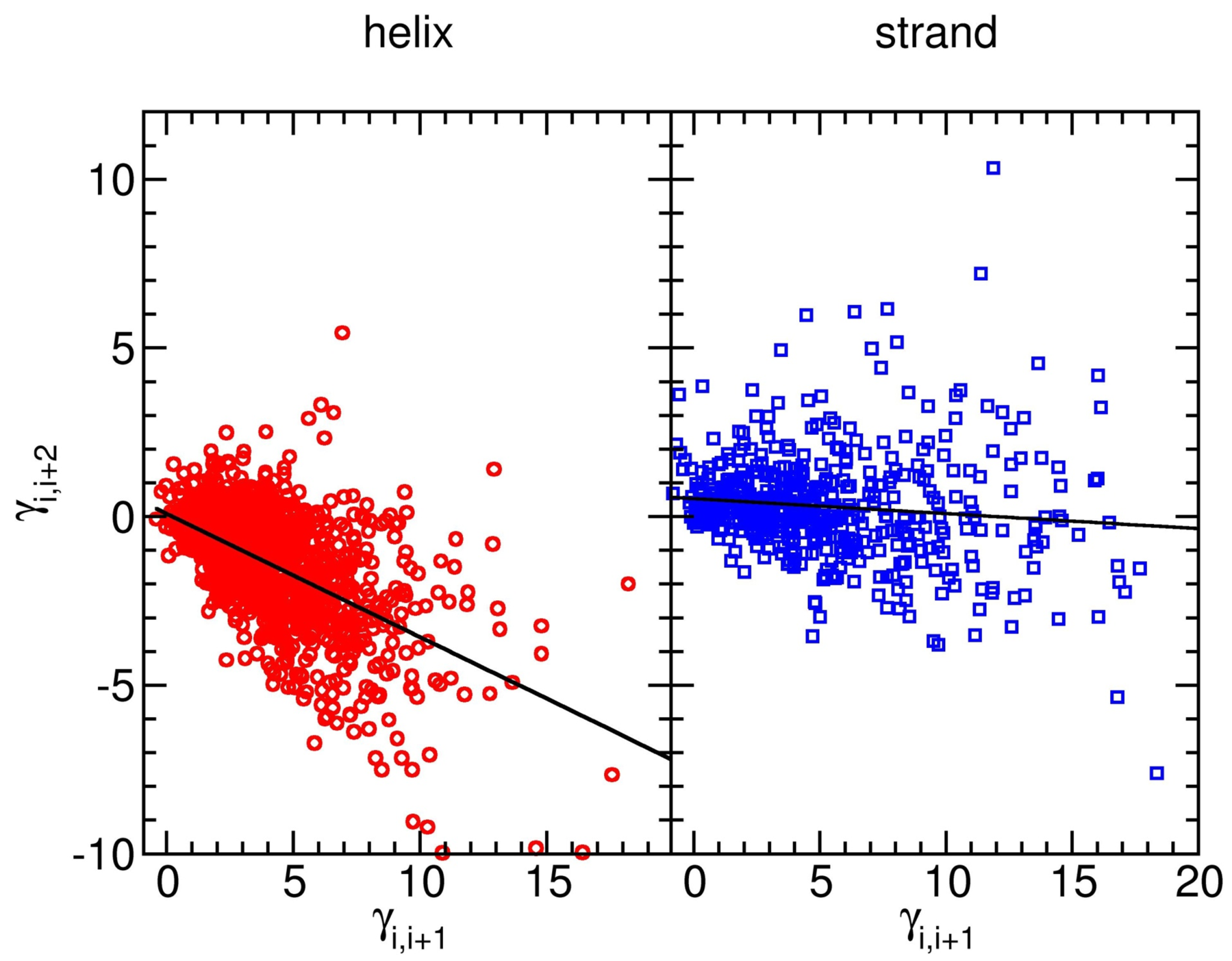

Supplement: Figure S2 — Scatter plots of k = 2 force constants against k = 1 force constants for helices (red circles) and strands (blue squares). (1.31 MB TIF) [file pcbi.1000816.s002.tif]

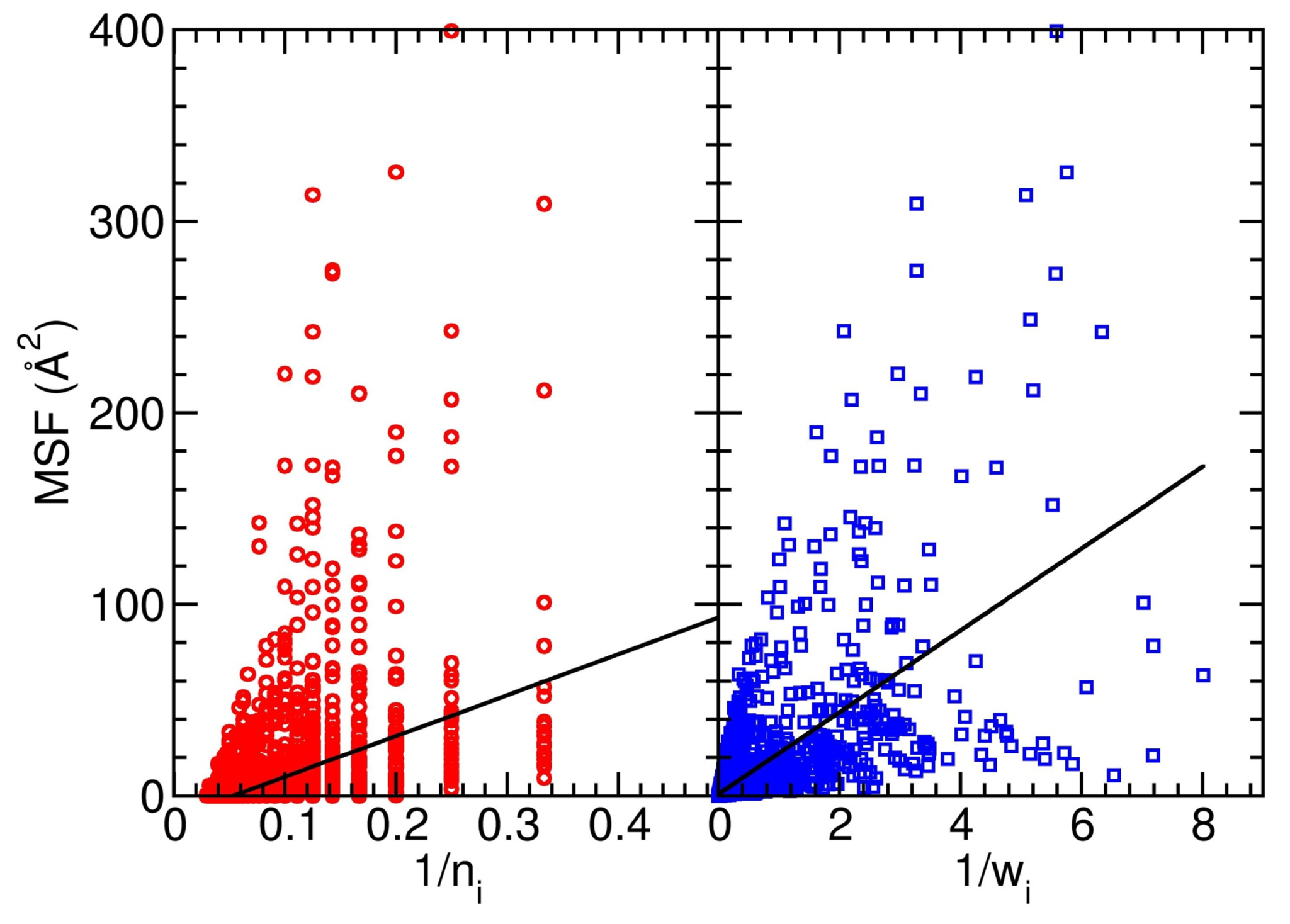

Supplement: Figure S3 — Relationship between mean square fluctuations and inverse node weight. In GNM (red circles) the weight of a node is the number of its edges, ni. In OFC-GNM (blue squares), the edge weight is the sum of the magnitudes of all its edges. The correlations with the linear fits shown are 0.416 and 0.670, respectively. (1.15 MB TIF) [file pcbi.1000816.s003.tif]
