# Supplementary material for: Using Entropy Maximization to Understand the Determinants of Structural Dynamics beyond Native Contact Topology
Source: PLoS Comput Biol. 2010 Jun 17;6(6):e1000816. doi: 10.1371/journal.pcbi.1000816 (PMC2887458; doi:10.1371/journal.pcbi.1000816)
Supplement: Table S1 — Training set proteins (0.06 MB DOC) [file pcbi.1000816.s004.doc]

**Table S1: Protein set**

| PDB ID | Number of Residues | Number of NMR models | PDB ID | Number of Residues | Number of NMR models |
| --- | --- | --- | --- | --- | --- |
| 2bds | 43 | 42 | 1faf | 79 | 47 |
| 1hyk | 46 | 40 | 1nhn | 79 | 41 |
| 1ncs | 47 | 46 | 1o8t | 79 | 68 |
| 1iox | 50 | 40 | 1xu6 | 80 | 60 |
| 1afp | 51 | 40 | 2no8 | 85 | 60 |
| 1bal | 51 | 56 | 1a6x | 87 | 49 |
| 2jv1 | 51 | 50 | 1bnp | 87 | 55 |
| 1mbf | 52 | 50 | 1emw | 88 | 47 |
| 1mbh | 52 | 50 | 1t4n | 88 | 51 |
| 1mbk | 52 | 50 | 1ap4 | 89 | 40 |
| 2p6j | 52 | 43 | 1iu2 | 91 | 50 |
| 1zrp | 53 | 40 | 1qo6 | 101 | 55 |
| 1yuj | 54 | 50 | 2ezn | 101 | 40 |
| 1gb1 | 56 | 60 | 1bpv | 104 | 50 |
| 1omt | 56 | 50 | 1hko | 104 | 42 |
| 1omu | 56 | 50 | 2giw | 104 | 40 |
| 1e9t | 59 | 85 | 2jps | 105 | 45 |
| 1ks0 | 59 | 50 | 1mph | 106 | 50 |
| 1cxw | 60 | 50 | 1wjb | 110 | 40 |
| 1j7m | 60 | 50 | 2jop | 110 | 50 |
| 2vrd | 61 | 47 | 2jp0 | 110 | 50 |
| 2jmd | 63 | 50 | 1j5h | 113 | 44 |
| 3gcc | 63 | 46 | 1o5p | 113 | 60 |
| 1c5a | 65 | 41 | 2g0l | 113 | 58 |
| 2jrb | 65 | 40 | 1fht | 116 | 43 |
| 1bw5 | 66 | 50 | 1h0t | 116 | 40 |
| 1r48 | 66 | 51 | 1myo | 118 | 44 |
| 1aww | 67 | 42 | 1mek | 120 | 40 |
| 1fh3 | 67 | 42 | 2bo5 | 120 | 44 |
| 1vig | 71 | 40 | 1cmo | 127 | 43 |
| 1iml | 76 | 48 | 2tmp | 127 | 49 |
| 1xqq | 76 | 128 | 1cey | 128 | 46 |
| 2nr2 | 76 | 144 | 2hm8 | 136 | 49 |
| 1uta | 77 | 45 | 1xwe | 151 | 40 |
