# Supplementary material for: Using Entropy Maximization to Understand the Determinants of Structural Dynamics beyond Native Contact Topology
Source: PLoS Comput Biol. 2010 Jun 17;6(6):e1000816. doi: 10.1371/journal.pcbi.1000816 (PMC2887458; doi:10.1371/journal.pcbi.1000816)
Supplement: Table S2 — Test set proteins (0.05 MB DOC) [file pcbi.1000816.s005.doc]

**Table S2: Test set**

| PDB ID | Number of Residues | Number of NMR models | PDB ID | Number of Residues | Number of NMR models |
| --- | --- | --- | --- | --- | --- |
| 1brz | 53 | 43 | 1rck | 105 | 42 |
| 1bbo | 56 | 60 | 1tru | 105 | 40 |
| 1fcl | 56 | 40 | 1trv | 105 | 40 |
| 1fd6 | 57 | 40 | 1te4 | 111 | 40 |
| 1gb4 | 57 | 47 | 1j5i | 113 | 44 |
| 2b88 | 58 | 40 | 2g0k | 113 | 47 |
| 2b89 | 58 | 40 | 1e5g | 120 | 50 |
| 1b3c | 64 | 40 | 1e8l | 129 | 50 |
| 2kdp | 71 | 47 | 1nwv | 129 | 42 |
| 3nla | 73 | 40 | 2a55 | 133 | 40 |
| 2k39 | 76 | 116 | 1c89 | 134 | 40 |
| 2kn5 | 76 | 50 | 2k9a | 136 | 50 |
| 1hsn | 79 | 49 | 2nef | 136 | 40 |
| 1r2u | 89 | 40 | 2kgf | 140 | 50 |
| 1r6p | 89 | 40 | 2k0e | 148 | 160 |
| 1spy | 89 | 40 | 2wcy | 155 | 48 |
| 1skt | 90 | 40 | 2k8v | 157 | 40 |
| 1smg | 90 | 40 | 2ke5 | 174 | 50 |
| 1tnp | 90 | 40 | 2k7p | 188 | 40 |
| 1tnq | 90 | 40 | 2jrc | 191 | 40 |
| 1do9 | 94 | 40 |  |  |  |
