# Supplementary material for: Using Entropy Maximization to Understand the Determinants of Structural Dynamics beyond Native Contact Topology
Source: PLoS Comput Biol. 2010 Jun 17;6(6):e1000816. doi: 10.1371/journal.pcbi.1000816 (PMC2887458; doi:10.1371/journal.pcbi.1000816)
Supplement: Table S3 — Test set mGNM results by protein (0.08 MB DOC) [file pcbi.1000816.s006.doc]

**Table S3: Test Set Results Columns show correlations between covariances determined from NMR ensembles and theoretically determined covariances using GNM and mGNM**

| **PDB** | **GNM** | | | **mGNM** | | |
| --- | --- | --- | --- | --- | --- | --- |
|  | **cc(RMSF)** | **cc(Off-Diag.)** | **cc(All)** | **cc(RMSF)** | **cc(Off-Diag)** | **cc(All)** |
| 1b3c | 6.931579e-01 | 4.510024e-01 | 6.896407e-01 | 7.172572e-01 | 5.584679e-01 | 6.996636e-01 |
| 1bbo | 8.870461e-01 | 3.968338e-01 | 5.290794e-01 | 8.007849e-01 | 4.150146e-01 | 4.667880e-01 |
| 1brz | 8.279314e-01 | 3.234734e-01 | 6.472535e-01 | 8.657504e-01 | 5.068461e-01 | 6.753830e-01 |
| 1c89 | 7.250430e-01 | 2.450390e-01 | 3.505609e-01 | 7.033544e-01 | 2.684802e-01 | 3.414933e-01 |
| 1do9 | 8.166116e-01 | 5.655332e-01 | 6.146646e-01 | 9.172397e-01 | 5.642006e-01 | 6.496918e-01 |
| 1e5g | 6.858609e-01 | 2.282661e-01 | 3.034650e-01 | 6.221754e-01 | 2.404570e-01 | 2.920779e-01 |
| 1e8l | 4.640129e-01 | 2.581471e-01 | 4.558704e-01 | 5.379836e-01 | 3.099417e-01 | 4.542841e-01 |
| 1fcl | 2.530804e-01 | 6.092143e-02 | 2.947633e-01 | 4.483992e-01 | 1.855543e-01 | 3.076553e-01 |
| 1fd6 | 5.475471e-01 | 3.800932e-01 | 6.623110e-01 | 5.076263e-01 | 4.923643e-01 | 6.470189e-01 |
| 1gb4 | 4.340194e-01 | 1.748118e-01 | 5.476197e-01 | 5.075261e-01 | 3.087041e-01 | 5.447579e-01 |
| 1hsn | 7.506672e-01 | 2.688470e-01 | 4.055339e-01 | 8.372991e-01 | 3.952433e-01 | 4.862300e-01 |
| 1j5i | 5.909267e-01 | 2.271530e-01 | 4.900941e-01 | 6.224309e-01 | 3.181154e-01 | 4.422386e-01 |
| 1nwv | 4.317864e-01 | 3.057546e-01 | 3.558786e-01 | 3.280965e-01 | 3.202020e-01 | 3.566701e-01 |
| 1r2u | 8.055436e-01 | 4.086349e-01 | 5.724839e-01 | 9.019656e-01 | 5.273874e-01 | 6.313446e-01 |
| 1r6p | 6.680844e-01 | 4.455564e-01 | 5.456523e-01 | 7.271267e-01 | 5.230682e-01 | 5.672173e-01 |
| 1rck | 6.450231e-01 | 3.352956e-01 | 6.335955e-01 | 7.338676e-01 | 4.902209e-01 | 6.473870e-01 |
| 1skt | 4.284693e-01 | 2.651199e-01 | 4.837270e-01 | 6.083265e-01 | 3.329506e-01 | 4.908799e-01 |
| 1smg | 8.896446e-01 | 4.772550e-01 | 6.563584e-01 | 9.369429e-01 | 6.265001e-01 | 7.136750e-01 |
| 1spy | 7.945976e-01 | 3.580116e-01 | 5.346801e-01 | 8.711287e-01 | 4.601500e-01 | 5.700274e-01 |
| 1te4 | 7.770084e-01 | 4.207371e-01 | 5.085447e-01 | 8.968105e-01 | 4.742289e-01 | 5.653549e-01 |
| 1tnp | 9.059351e-01 | 4.243846e-01 | 6.350396e-01 | 9.036128e-01 | 5.125971e-01 | 5.972692e-01 |
| 1tnq | 7.752112e-01 | 4.245057e-01 | 5.627860e-01 | 8.835354e-01 | 5.232206e-01 | 5.941132e-01 |
| 1tru | 6.075458e-01 | 4.873217e-01 | 6.619645e-01 | 5.252736e-01 | 5.596850e-01 | 6.853586e-01 |
| 1trv | 4.642164e-01 | 3.702009e-01 | 5.799997e-01 | 4.355759e-01 | 4.636409e-01 | 6.011281e-01 |
| 2a55 | 8.381598e-01 | 2.370470e-01 | 3.496981e-01 | 8.521773e-01 | 2.733400e-01 | 3.391852e-01 |
| 2b88 | 8.808518e-01 | 5.492774e-01 | 6.994432e-01 | 9.098134e-01 | 5.603378e-01 | 7.049459e-01 |
| 2b89 | 5.735574e-01 | 1.745616e-01 | 4.269976e-01 | 7.659740e-01 | 3.446088e-01 | 5.256993e-01 |
| 2g0k | 6.473511e-01 | 4.363477e-01 | 5.970675e-01 | 6.614950e-01 | 4.938705e-01 | 6.047468e-01 |
| 2jrc | 7.052411e-01 | 4.586164e-01 | 5.791268e-01 | 7.747398e-01 | 5.159443e-01 | 6.017787e-01 |
| 2k0e | 5.426294e-01 | 1.859663e-01 | 2.602017e-01 | 5.080561e-01 | 1.815932e-01 | 2.308924e-01 |
| 2k39 | 5.870685e-01 | 3.535569e-01 | 4.159642e-01 | 7.703773e-01 | 4.366664e-01 | 4.988923e-01 |
| 2k7p | 9.696187e-01 | 7.580932e-01 | 7.928299e-01 | 9.718054e-01 | 7.359836e-01 | 7.767406e-01 |
| 2k8v | 9.547234e-01 | 8.462796e-01 | 8.297932e-01 | 9.811549e-01 | 8.452710e-01 | 8.680942e-01 |
| 2k9a | 9.323516e-01 | 7.495486e-01 | 7.745154e-01 | 9.723575e-01 | 7.726053e-01 | 8.108811e-01 |
| 2kdp | 8.854471e-01 | 5.817704e-01 | 6.826764e-01 | 9.175880e-01 | 5.702457e-01 | 6.790681e-01 |
| 2ke5 | 4.432587e-01 | 4.242679e-01 | 4.760453e-01 | 5.824319e-01 | 5.285592e-01 | 5.107333e-01 |
| 2kgf | 6.323099e-01 | 5.839211e-01 | 6.085068e-01 | 7.633952e-01 | 6.260473e-01 | 6.587202e-01 |
| 2kn5 | 2.195412e-01 | 3.594121e-01 | 5.860188e-01 | 2.348255e-01 | 4.428505e-01 | 5.833492e-01 |
| 2nef | 8.416216e-01 | 4.666471e-01 | 5.902496e-01 | 8.983138e-01 | 5.184157e-01 | 6.144454e-01 |
| 2wcy | 8.719235e-01 | 5.908000e-01 | 6.434386e-01 | 9.171870e-01 | 6.217366e-01 | 6.779715e-01 |
| 3nla | 8.367809e-01 | 4.340358e-01 | 6.436534e-01 | 9.193401e-01 | 5.218778e-01 | 6.519890e-01 |
